# Comparative effectiveness of adding delamanid to a multidrug-resistant tuberculosis regimen comprised of three drugs likely to be effective

**DOI:** 10.1371/journal.pgph.0000818

**Published:** 2023-04-28

**Authors:** Carly A. Rodriguez, Sara Lodi, C. Robert Horsburgh, Carole D. Mitnick, Mathieu Bastard, Helena Huerga, Uzma Khan, Michael Rich, Kwonjune J. Seung, Sidney Atwood, Md Manzur-ul-Alam, Nara Melikyan, Stephanie Mpinda, Zaw Myint, Yugandran Naidoo, Ofelya Petrosyan, Naseem Salahuddin, Samreen Sarfaraz, Stalz Charles Vilbrun, Kalkidan Yae, Jay Achar, Saman Ahmed, Elena Algozhina, Jude Beauchamp, Sara de Guadelupe Perea Moreno, Munara Gulanbaeva, Marika Gergedava, Cut Yulia Indah Sari, Catherine Hewison, Palwasha Khan, Molly F. Franke

**Affiliations:** 1 Department of Epidemiology, Boston University School of Public Health, Boston, Massachusetts, United States of America; 2 Department of Global Health and Social Medicine, Harvard Medical School, Boston, Massachusetts, United States of America; 3 Department of Biostatistics, Boston University School of Public Health, Boston, Massachusetts, United States of America; 4 Department of Global Health, Boston University School of Public Health, Boston, Massachusetts, United States of America; 5 Department of Medicine, Boston University School of Medicine, Boston, Massachusetts, United States of America; 6 Partners In Health, Boston, Massachusetts, United States of America; 7 Division of Global Health Equity, Brigham and Women’s Hospital, Boston, Massachusetts, United States of America; 8 Epicentre, Paris, France; 9 Interactive Research and Development Global, Singapore, Singapore; 10 Interactive Research and Development, Dhaka, Bangladesh; 11 Partners In Health, Maseru, Lesotho; 12 National Tuberculosis Program, Ministry of Health, Yangon, Myanmar; 13 Interactive Research and Development, Johannesburg, South Africa; 14 Médecins Sans Frontières, Yerevan, Armenia; 15 Indus Hospital & Health Network (IHHN), Karachi, Pakistan; 16 GHESKIO, Port-au-Prince, Haiti; 17 Partners In Health, Addis Ababa, Ethiopia; 18 Médecins Sans Frontières, United Kingdom; 19 Interactive Research and Development, Karachi, Pakistan; 20 Partners In Health, Almaty Kazakhstan; 21 Zanmi Lasante, Cange, Haiti; 22 Socios En Salud Sucursal, Lima, Peru; 23 Médecins Sans Frontières, Osh, Kyrgyzstan; 24 Médecins Sans Frontières, Tbilisi, Georgia; 25 Rumah Sakit Islam Jakarta Cempaka Putih, Jakarta, Indonesia; 26 Médecins Sans Frontières, Paris, France; Translational Health Science and Technology Institute, INDIA

## Abstract

Clarity about the role of delamanid in longer regimens for multidrug-resistant TB is needed after discordant Phase IIb and Phase III randomized controlled trial results. The Phase IIb trial found that the addition of delamanid to a background regimen hastened culture conversion; the results of the Phase III trial were equivocal. We evaluated the effect of adding delamanid for 24 weeks to three-drug MDR/RR-TB regimens on two- and six-month culture conversion in the endTB observational study. We used pooled logistic regression to estimate the observational analogue of the intention-to-treat effect (aITT) adjusting for baseline confounders and to estimate the observational analogue of the per-protocol effect (aPP) using inverse probability of censoring weighting to control for time-varying confounding. At treatment initiation, 362 patients received three likely effective drugs (delamanid-free) or three likely effective drugs plus delamanid (delamanid-containing). Over 80% of patients received two to three Group A drugs (bedaquiline, linezolid, moxifloxacin/levofloxacin) in their regimen. We found no evidence the addition of delamanid to a three-drug regimen increased two-month (aITT relative risk: 0.90 (95% CI: 0.73–1.11), aPP relative risk: 0.89 (95% CI: 0.66–1.21)) or six-month culture conversion (aITT relative risk: 0.94 (95% CI: 0.84, 1.02), aPP relative risk: 0.93 (95% CI: 0.83, 1.04)). In regimens containing combinations of three likely effective, highly active anti-TB drugs the addition of delamanid had no discernible effect on culture conversion at two or six months. As the standard of care for MDR/RR-TB treatment becomes more potent, it may become increasingly difficult to detect the benefit of adding a single agent to standard of care MDR/RR-TB regimens. Novel approaches like those implemented may help account for background regimens and establish effectiveness of new chemical entities.

## Introduction

An estimated 10 million people globally developed tuberculosis (TB) disease in 2021 [1]. Of these, nearly 500,000 became sick with a strain resistant to at least rifampin (rifampin resistant TB, RR-TB), or rifampin and isoniazid (multidrug-resistant TB, MDR-TB), the most effective first-line drugs to treat TB [2]. World Health Organization (WHO) guidance on treatment for MDR/RR-TB at the time of study conduct recommended a shorter (9–11 month) seven-drug regimen and longer (18–24 month) regimens of at least four drugs likely to be effective. Many of the drugs that comprise these regimens cause debilitating side effects [3–5].

The introduction of delamanid (OPC-67683, Deltyba)—one of the first drugs with a novel mechanism of action against *M*. *tuberculosis* in nearly 50 years—offers the potential to improve treatment for RR/MDR-TB. In 2014 the European Medicines Agency (EMA) conditionally approved delamanid for TB [6]. Results of the Phase IIb randomized controlled trial (RCT) (ClinicalTrials.gov identifier NCT00685360) showed delamanid to be efficacious: at two months, 45.4% of patients in the delamanid-plus-standard treatment arm experienced culture conversion vs 29.6% in the standard treatment arm (p = 0.008) [7]. However, the Phase III trial (ClinicalTrials.gov identifier NCT01424670) did not demonstrate a clinically relevant or statistically significant difference: 87.6% in the delamanid-plus-standard treatment arm experienced conversion by six months vs 86.1% in the placebo-plus-standard treatment arm [8].

One major difference between the trials was the composition of the background regimen. Phase II trial participants received four to five drugs on average, as compared to a mean of 6.5 drugs in Phase III trial. And, Phase III trial regimens tended to be more potent, which could have potentially masked delamanid’s contribution to treatment outcomes. The conflicting findings and different treatment regimens used across these trials beg for further investigation of delamanid’s role in MDR-TB regimens.

Based on the Phase III trial results, delamanid was categorized as a lower priority drug, to be used if a regimen with more effective drugs cannot be composed (e.g., resistance, intolerability) [9, 10]. There have also been calls for studies of delamanid in regimens compromised by resistance or intolerability, two features often resulting in patients receiving too few effective drugs [11]. However, evidence to date has not been from robust comparative effectiveness studies. In an early descriptive report of 66 patients with limited treatment options receiving delamanid under compassionate use, 80% were culture negative at six months [12]. Patients had, on average received 3.3 likely effective drugs. This treatment response—which far exceeded that from historical cohorts treated without delamanid—suggested delamanid may be beneficial for patients on few drugs. Subsequent descriptive studies of patients treated with delamanid showed similar early success, with 70–95% of patients achieving conversion within six months of delamanid initiation [13–17]. However, owing to a lack of comparative studies, the role of delamanid when added to a regimen containing fewer than four drugs, the number recommended by WHO for longer individualized treatment, remains an open question.

Here, using a robust analytic design and methods grounded in causal inference, we evaluated the comparative effect on two- and six-month culture conversion of adding versus not adding delamanid for 24 weeks to MDR-TB regimens comprising only three likely effective drugs instead of the four recommended by WHO at the time of the study.

## Materials and methods

### Data source and study population

We used data from the endTB observational cohort (ClinicalTrials.gov identifier NCT03259269), a prospective research cohort across 17 countries, and included participants with a positive culture and documented MDR/RR-TB at enrollment. Full details on the study protocol have been published previously [18]. In summary, participants were treated under routine programmatic conditions with a longer multidrug regimen including bedaquiline and/or delamanid, in accordance with guidelines of their respective countries and of WHO during the study period (2015–2020) [19, 20]. Clinical care was further informed by the endTB clinical guide [21]. Research activities were directed by a common protocol [18]. Data were collected using standardized forms and adverse events were monitored through a unified pharmacovigilance system [22].

### Design of comparative effectiveness analysis

We designed our analysis using target trial emulation to answer the causal question of interest: [23–28] what is the comparative effectiveness of adding delamanid for 24 weeks to an MDR-TB regimen of three drugs likely to be effective? We first specified a hypothetical, pragmatic “target” RCT (Appendix A in S1 Text). We then emulated this target trial with our observational data and conducted a statistical analysis to control for potential biases.

### Outcome

Culture conversion is used as an interim microbiological indicator and surrogate endpoint in both observational studies and RCTs [29, 30]. We assessed two- and six-month culture conversion risks. We defined culture conversion as the first of two consecutive negative cultures collected ≥15 days apart. Participants who died or were lost to follow-up (LTFU) before conversion were considered as not having converted. LTFU was defined as treatment interruption (i.e. no treatment) for ≥2 months.

### Eligibility criteria

We included participants with a positive baseline sputum culture, defined as any culture on a sputum specimen collected ≤90 days before treatment initiation in the endTB cohort [31, 32]. We excluded patients treated in the Democratic People’s Republic of Korea due to substantial differences in diagnosis, treatment delivery, and lack of HIV testing, compared to the rest of the cohort. Likely effectiveness of a drug was considered established if: (1) resistance testing indicated the participant’s *M*.*tb* strain was not resistant to the drug, or (2) no resistance testing had been conducted and the participant had not previously received the drug for one month or more, according to the medical record. All drugs in the WHO hierarchy were considered. Baseline regimens (i.e., those prescribed at the end of first week of treatment) were categorized as follows, based on the number of likely effective drugs and irrespective of WHO drug group hierarchy:[5] (1) receiving a regimen of delamanid plus a background regimen of three likely effective drugs, (2) receiving a regimen of exactly three likely effective drugs, none of which was delamanid, or (3) neither 1 or 2 (excluded from analyses). A three-drug regimen was used as the comparator because such regimens did not conform to WHO recommendations of the time, and we hypothesized that, among patients receiving three-drug regimens, culture conversion could be hastened with delamanid. In this cohort, a patient may have received a regimen of three likely effective drugs when other options were not available, because of high drug resistance, adverse events, or unavailability of drugs. Applying these criteria resulted in regimens primarily comprised of bedaquiline, linezolid, levofloxacin/moxifloxacin, and clofazimine.

### Statistical analysis

We estimated the observational analogue of the intention-to-treat (aITT) effect and the observational analogue of the per-protocol effect (aPP). The analysis estimating the aITT effect includes all participants, classified by their baseline treatment, and adjusted for baseline confounders. This analysis estimates the effect of initiating delamanid plus a background regimen of three drugs versus initiating a background regimen of three drugs, not including delamanid. Treatment could change during follow-up. For some participants in the delamanid-containing group, delamanid was discontinued; for some participants in the delamanid-free group, delamanid was started; and in both groups, some patients experienced changes in the number of background drugs. The aPP effect estimates the effect of adding and remaining on delamanid among participants who received a regimen of three likely effective drugs for the duration of follow-up (up to 24 weeks). Because MDR-TB treatment can vary over time, aPP analyses may be biased by time-dependent confounding [33–35].

#### Estimating the intention-to-treat analogue relative risk and risk difference of culture conversion

We fitted a pooled logistic regression model and its predicted probabilities to estimate the risk of conversion for delamanid-containing versus delamanid-free regimens. The model was adjusted for the following baseline confounders chosen a priori using content knowledge and directed acyclic graphs: age, sex, hospitalization at treatment initiation, the number of Group A drugs (i.e., those classified as priority drugs in the 2020 WHO MDR/RR-TB guidelines including bedaquiline, linezolid, moxifloxacin/levofloxacin), whether the patient was receiving imipenem-cilastatin, body mass index <18.5, HIV infection, and hepatitis C antibody positivity. Confidence intervals were estimated using nonparametric bootstrapping with 500 samples [36]. Missing data were rare for most confounders (<1%), with the exception of baseline cavitation on chest radiography (Table 1). Primary analyses were complete case.

**Table 1:** Baseline characteristics of participants receiving three drugs likely to be effective +/- delamanid, endTB observational cohort (N = 362).

	Treatment group				Overall, N = 362	
	DLM-containing, n = 123		DLM-free, n = 239			
	n	(%)	N	(%)	N	(%)
Site						
Armenia	11	(8.9)	11	(4.6)	22	(6.1)
Bangladesh	9	(7.3)	25	(10.5)	34	(9.4)
Belarus	15	(12.2)	2	(0.8)	17	(4.7)
Georgia	12	(9.8)	49	(20.5)	61	(16.9)
Haiti	5	(4.1)	0	0	5	(1.4)
Indonesia	0	0	1	(0.4)	1	(0.3)
Kazakhstan	53	(43.1)	56	(23.4)	109	(30.1)
Kyrgyzstan	2	(1.6)	2	(0.8)	4	(1.1)
Lesotho	3	(2.4)	5	(2.1)	8	(2.2)
Myanmar	3	(2.4)	0	0	3	(0.8)
Pakistan	9	(7.3)	35	(14.6)	44	(12.2)
Peru	0	0	51	(21.3)	51	(14.1)
South Africa	1	(0.8)	0	0	1	(0.3)
Vietnam	0	0	2	(0.8)	2	(0.6)
Age, median (SD)	39	(11.6)	35	(12.6)	36	(12.3)
Male	80	(64.0)	166	(69.7)	246	(67.8)
Baseline cavity						
Missing	6	(4.9)	33	(13.8)	39	(10.8)
Cavitation	90	(73.2)	141	(59.0)	231	(63.8)
No cavitation	27	(22.0)	65	(27.2)	92	(25.4)
Bilateral disease						
Missing	3	(2.4)	26	(10.9)	29	(8.0)
Bilateral	87	(70.7)	146	(61.1)	233	(64.4)
Non-bilateral	33	(26.8)	67	(28.0)	100	(27.6)
Smear grade (-90 days, +0 days from treatment initiation)						
Missing	6	(4.9)	5	(2.1)	11	(3.0)
Negative	27	(22.0)	65	(27.2)	92	(25.4)
Scanty 1–3	3	(2.4)	2	(0.8)	5	(1.4)
Scanty 4–9	4	(3.3)	8	(3.3)	12	(3.3)
One plus	44	(35.8)	77	(32.2)	121	(33.4)
Two plus	16	(13.0)	37	(15.5)	53	(14.6)
Three plus	22	(17.9)	44	(18.4)	66	(18.2)
Disease site						
Extrapulmonary	0	0	3	(1.3)	3	(0.8)
Pulmonary	123	(100.0)	236	(98.7)	359	(99.2)
HIV						
Missing	1	(0.8)	0	0	1	(0.3)
Negative	105	(85.4)	228	(95.4)	333	(92.0)
Positive	17	(13.8)	11	(4.6)	28	(7.7)
Hepatitis B infection						
Missing	1	(0.8)	0	0	1	(0.3)
Negative	114	(92.7)	232	(97.1)	346	(95.6)
Positive	8	(6.5)	7	(2.9)	15	(4.1)
Hepatitis C infection						
Missing	2	(1.6)	0	0	2	(0.6)
Negative	94	(76.4)	212	(88.7)	306	(84.5)
Positive	27	(22.0)	27	(11.3)	54	(14.9)
Malnutrition (body mass index <18.5)						
Missing	2	(1.6)	0	0	2	(0.6)
No malnutrition	71	(57.7)	156	(65.3)	227	(62.7)
Malnutrition	50	(40.7)	83	(34.7)	133	(36.7)
Diabetes						
Missing	1	(0.8)	3	(1.3)	4	(1.1)
No diabetes	95	(77.2)	209	(87.4)	304	(84.0)
Diabetes	27	(22.0)	27	(11.3)	54	(14.9)
Previous TB treatment						
Missing	4	(3.3)	4	(1.7)	8	(2.2)
Only with first-line drugs	12	(9.8)	2	(0.8)	14	(3.9)
With second-line drugs	104	(84.6)	233	(97.5)	337	(93.1)
Number of Group A drugs*						
3 Group A drugs	0	0	24	(10.0)	24	(6.6)
2 Group A drugs	72	(58.5)	197	(82.4)	269	(74.3)
1 Group A drug	49	(39.8)	18	(7.5)	67	(18.5)
No Group A drugs	2	(1.6)	0	0	2	(0.6)

**Abbreviations:** standard deviation (SD), delamanid (DLM)

*Group A drugs include bedaquiline, linezolid, moxifloxacin/levofloxacin

#### Estimating the per-protocol analogue relative risk and risk difference of culture conversion

To estimate the aPP effect, we artificially censored participants when their treatment deviated from that administered at baseline. To adjust for selection bias due to this artificial censoring, we applied inverse probability of censoring weights [37].

To simulate the per-protocol population, we censored observations in the delamanid-containing group when delamanid had been discontinued for >2 consecutive weeks prior to the end of treatment and there was no evidence that delamanid discontinuation was in response to an adverse event. Therefore, estimated effects reflect continuation of delamanid for up to 24 weeks, unless contraindicated due to an adverse event. In the delamanid-free group, we censored observations when delamanid was added for >2 consecutive weeks. In both groups, observations were censored if drugs were added or removed from the regimen such that it contained either less than three or more than three drugs likely to be effective for >2 consecutive weeks. To control for selection bias due to artificial censoring, for each individual and for each week, we estimated time-varying inverse probability of censoring weights equal to the inverse of the probability of being uncensored, i.e. maintaining a regimen consistent with the baseline treatment group.

To estimate time-varying weights, we fitted a pooled logistic regression model to estimate the probability each participant remained on their baseline treatment (i.e., was not censored) conditional on time-varying predictors of changing treatment and time since baseline. These predictors included time-varying number of Group A drugs according to WHO classification, sputum smear result, number of adverse events, hospitalization, time, and a quadratic function of time (Primary Model, Appendix B in S1 Text). Full detail on the derivation of weights is provided in Appendix C in S1 Text.

Using a weighted logistic regression model adjusted for baseline confounders of treatment, we estimated the predicted probabilities of culture conversion for each uncensored participant. We then used the mean predicted probability of conversion by treatment group to calculate the point estimate for the relative risk and risk difference. Confidence intervals were calculated using nonparametric bootstrapping with 500 samples.

#### Sensitivity analyses

To account for the possibility that adjustment for the number of Group A drugs did not adequately control for the efficacy of the background regimen across treatment groups, we conducted a sensitivity analysis among patients who had received bedaquiline. We restricted the delamanid-containing group to those who also received bedaquiline.

### Ethical approval

We obtained approvals from the central ethics review committees for each consortium partner and local ethics committees in each country. This study was approved by the Partners Healthcare Human Research Committee (Boston, MA, USA), the MSF Ethics Review Board (Geneva, Switzerland), IRD Institutional Review Board (Karachi, Pakistan) and in all 17 implementing countries by appropriate government authorities or local ethics committees (Armenia: Ethics Committee of Yerevan State Medical University after Mkhitar Heratsi; Bangladesh: Ethical Committee, National Institute of Diseases of the Chest and Hospital; Belarus: Ethics Committees of the Republican Scientific and Practical Centre of Pulmonology and Tuberculosis; DPRK: Ministry of Public Health; Ethiopia: National Research Ethics Review Committee of Ministry of Scient and Technology; Georgia: Ethics Committee of National Center for Tuberculosis and Lung Diseases; Haiti: Comité Des Droits Humains Des Centres GHESKIO, Zanmi Lasante Research Committee; Indonesia: Ethics Committee, Faculty of Medicine, Universitas Indonesia; Kazakhstan: National Scientific Center of Phthisiopulmonology of the Ministry of Health; Kenya: The Scientific and Ethics Review Unit, Kenya Medical Research Institute; Kyrgyzstan: Committee on Bioethics under the MoH of the Kyrgyz Republic; Lesotho: Ministry of Health Research and Ethics Committee; Myanmar: Ethics Review Committee, Department of Medical Research, Ministry of Health and Sports; Pakistan: IRD Institutional Review Board; Peru: Institutional Research Ethics Committee at the Peruvian University of Cayetano Heredia; South Africa: Bio Medical Research Ethics Committee, University of KwaZulu-Natal; Vietnam: Sciences and Ethical Committee of the National Lung Hospital and Independent Ethics Committee, Ministry of Health). Participants provided written informed consent.

## Results

**Fig 1:**
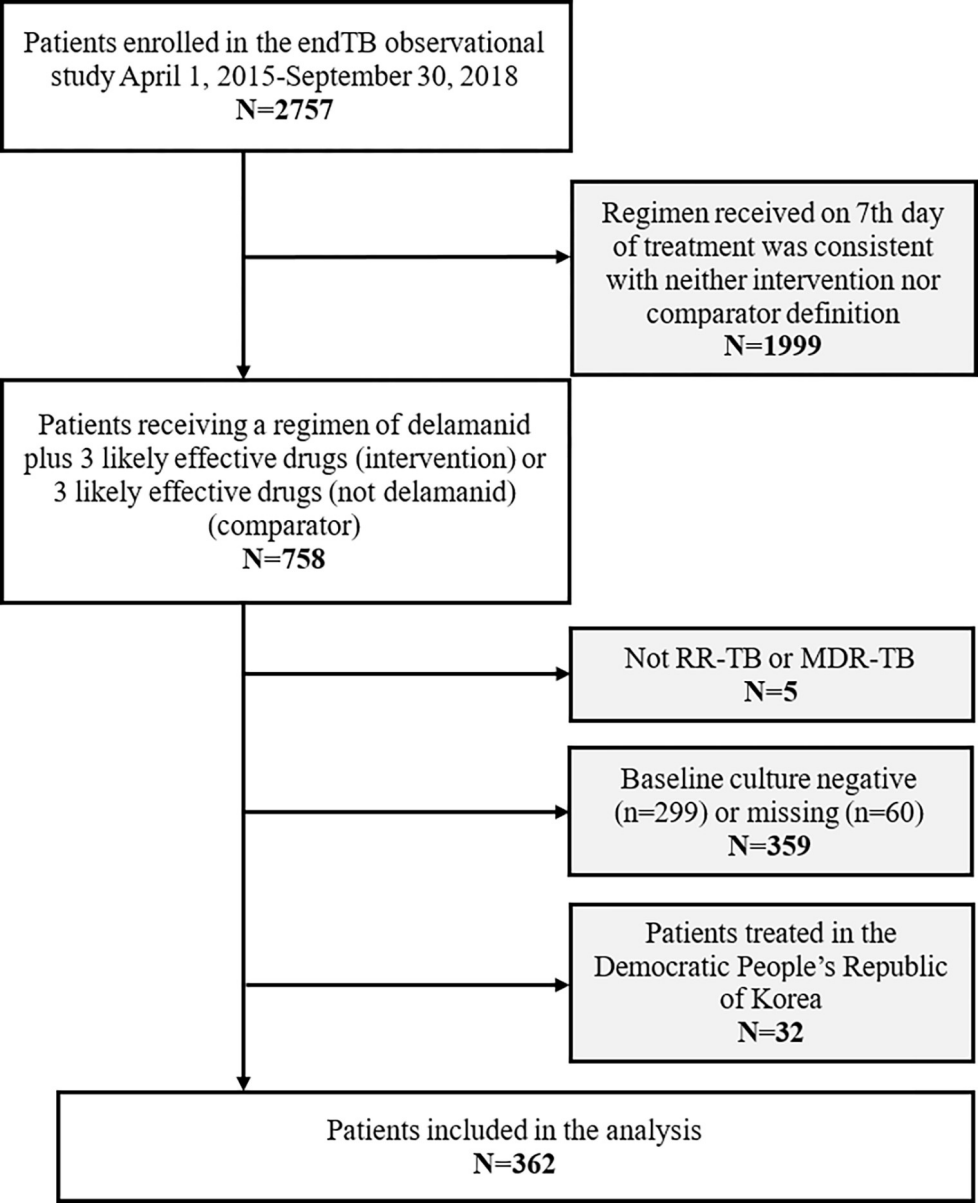
Flowchart of patients eligible for the comparative effectiveness of delamanid analysis in the endTB observational cohort.

Between April 1, 2015 and September 30, 2018, 2757 patients were initiated on a first MDR/RR regimen containing bedaquiline and/or delamanid and consented to participate in the endTB observational study (Fig 1). We excluded 1999 (72.5%) participants whose baseline regimen did not correspond to a treatment group of interest. Patients who did not have RR/MDR-TB (n = 5), had a negative or missing baseline sputum culture (n = 359), or were treated in the Democratic People’s Republic of North Korea (N = 32) were excluded, leaving 362 participants (N = 123 delamanid-containing, N = 239 delamanid-free) in the aITT cohort (Fig 1).

Most patients were treated in Kazakhstan (30.1%), Georgia (16.9%), Peru (14.1%), or Pakistan (12.2%) (Table 1). Treatment groups were comparable in age, sex, and indicators of disease severity such as cavitation, bilateral disease, and smear grade; however, missing data on cavitation and bilateral disease was more common for participants in the delamanid-free group. The delamanid-containing group had a greater proportion of participants with comorbidities (Table 1).

Although participants in both groups received a background regimen of three drugs likely to be effective, there was substantial heterogeneity in companion drugs (Appendix D in S1 Text). On average, participants in the delamanid-containing group had fewer Group A drugs. In the delamanid-free group, 10.0% of participants received all three Group A drugs and 82.4% received two Group A drugs. In the delamanid-containing group, no participant received all three Group A drugs and 58.5% received two Group A drugs (Table 1).

**Fig 2:**
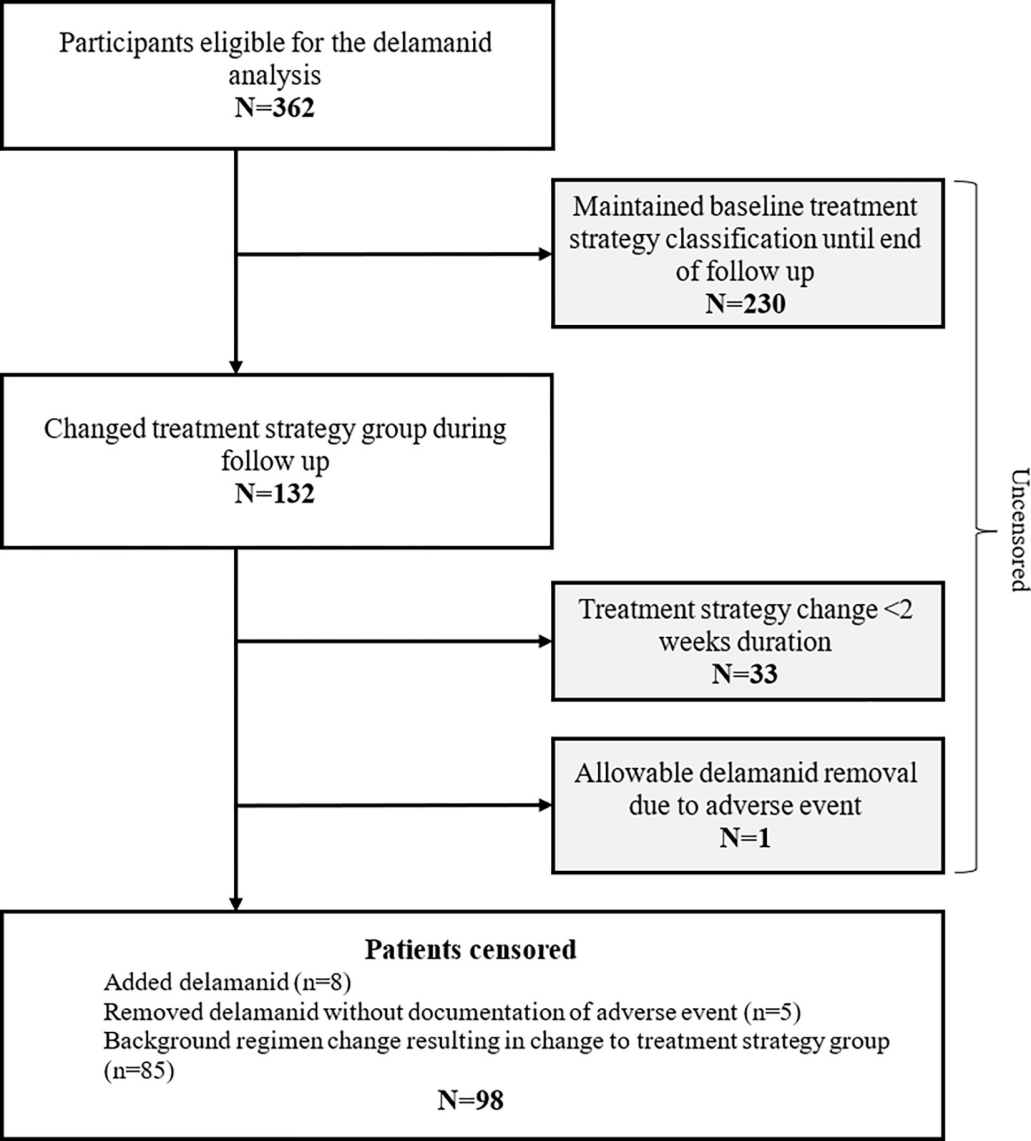
Flowchart of participants censored due to changing treatment strategies in the endTB observational cohort.

The baseline treatment regimen was maintained for 24 continuous weeks (Fig 2) in 63.5% of participants. Of the 132 whose treatment regimen changed, 33 were short term (<2 weeks). In one additional participant, delamanid was removed due to an adverse event. The remaining 98 participants had regimen adjustments that resulted in a treatment group change and censoring due to addition of delamanid (n = 8); delamanid withdrawal without a documented, related adverse event (n = 5); and a background regimen change (n = 85, Fig 2). The distribution of censoring weights is shown in Appendix B in S1 Text. Adjusted aPP estimates were calculated from 348 of 362 participants with complete data (Table 2).

**Table 2:** Effect of adding delamanid to a regimen composed of three drugs likely to be effective, endTB observational cohort (N = 362).

		Two-month culture conversion		Six-month culture conversion	
**Analysis**	**N**	**RR (95% CI)**	**RD (95% CI)**	**RR (95% CI)**	**RD (95% CI)**
Crude	362	0.89 (0.72, 1.10)	-0.06 (-0.17, 0.05)	0.92 (0.83, 1.01)	-0.07 (-0.15, -0.01)
*Intention-to-treat analogue (aITT)*					
Logistic regression	358^a^	0.90 (0.73, 1.11)	-0.05 (-0.16, 0.06)	0.94 (0.84, 1.02)	-0.06 (-0.14, 0.02)
*Per-protocol analogue (aPP)*					
IP censoring weighted, unstabilized	348^b^	0.89 (0.60, 1.30)	-0.06 (-0.26, 0.16)	0.93 (0.82, 1.05)	-0.06 (-0.17, 0.05)
IP censoring weighted, stabilized	348^b^	0.89 (0.66, 1.21)	-0.06 (-0.21, 0.11)	0.93 (0.83, 1.04)	-0.06 (-0.16, 0.03)
IP censoring weighted, stabilized, among patients on BDQ	288^c^	0.94 (0.54, 1.33)	-0.04 (-0.28, 0.17)	1.04 (0.91, 1.11)	0.04 (-0.08, 0.10)

**Abbreviations:** Inverse probability (IP), risk ratio (RR), risk difference (RD), bedaquiline (BDQ)

^a^ N = 4 participants excluded for missing baseline data

^b^ N = 10 participants excluded for missing time-varying data, N = 2 participants excluded for missing baseline and time-varying data, N = 2 participants excluded for missing baseline data

^c^ N = 7 participants excluded for missing time-varying data, N = 1 participant excluded for missing baseline data

Within two months, 49.6% of participants in the delamanid-containing group and 55.6% of participants in the delamanid-free group experienced culture conversion. Among participants who did not have culture conversion, 6 (4.8%) (3 deaths, 3 LTFU) participants in the delamanid-containing group and 7 (2.9%) (2 deaths, 5 LTFU) participants in the delamanid-free group were not retained in care. In aITT analyses, the RR of culture conversion for delamanid-containing versus delamanid-free regimens was 0.90 (95% CI: 0.73, 1.11) and RD was -0.05 (95% CI: -0.16, 0.06). In the aPP analysis the RR of culture conversion for delamanid-containing versus delamanid-free regimens was 0.89 (95% CI: 0.66, 1.21) and the RD was -0.06 (95% CI: -0.21, 0.11)) (Table 2).

By six months, 81.3% of participants in the delamanid-containing and 88.7% of participants in the delamanid-free group experienced sputum culture conversion. Fifteen (12.2%) (7 deaths, 8 LTFU) participants in the delamanid-containing group died or were LTFU before conversion versus 14 (5.9%) (4 deaths, 10 LTFU) in the delamanid-free group. In aITT analyses, the RR of culture conversion for delamanid-containing versus delamanid-free regimens was 0.94 (95% CI: 0.84, 1.02) and the RD was -0.06 (95% CI: -0.14, 0.02). In the aPP analysis the RR of culture conversion for delamanid-containing versus delamanid-free regimens was 0.93 (95% CI: 0.83, 1.04) and RD was -0.06 (95% CI: -0.16, 0.03) (Table 2).

When we restricted analyses to participants receiving bedaquiline in their baseline regimen, the two-month aPP RR of culture conversion suggested comparable frequencies, with an RR of 0.94 (95% CI: 0.54, 1.33) and RD of -0.04 (95% CI: -0.28, 0.17) (Table 2). Estimates for six-month culture conversion were similar, with an RR of 1.04 (95% CI: 0.91, 1.11) and RD of 0.04 (95% CI: -0.08, 0.10) (Table 2).

## Discussion

In the context of regimens containing exactly three likely effective drugs, many of which comprised at least two Group A drugs and clofazimine, we found no evidence of an effect of adding delamanid on culture conversion within two and six months. This finding is consistent with the previously reported Phase III delamanid trial but different from that of the Phase IIb trial [7, 8]. A major strength of our study is that we collected and used longitudinal data on treatment changes and time-varying risk factors that have historically not been represented in observational TB cohorts [38]. This, in combination with an approach rooted in target trial emulation, allowed us to answer a precise comparative effectiveness question and account for potential biases that could not be addressed in prior observational studies of MDR/RR treatment.

Our treatment groups were defined by the *quantity* of drugs in a regimen. However, the efficacy of drugs in the regimen cannot be ignored. The advent of bedaquiline, repurposed drugs, such as linezolid and clofazimine, and late generation quinolones, such as levofloxacin/moxifloxacin, have transformed the TB treatment landscape. Regimens comprised of drugs with lesser efficacy, like those used in the Phase IIb trial that showed a significant effect of delamanid, [7] are not represented in large numbers in the endTB cohort. For example, in the Phase IIb trial, many patients received pyrazinamide, kanamycin, cycloserine, and ethambutol. In contrast, regimens in our study included bedaquiline, and made even greater use of other potent drugs like linezolid and moxifloxacin/levofloxacin than did the Phase III trial, [8, 39] which, similarly, found no effect of delamanid on median time to culture conversion over six months. Thus, it is not surprising our findings resemble those from the latter study. Whether delamanid can improve the effectiveness of regimens compromised by toxicity or resistance to Group A drugs, or prevent acquired resistance through protection of these drugs, remains unanswered.

Future MDR-TB treatment research aimed at comparative effectiveness should examine both the number and efficacy of drugs in a regimen. Delamanid-free regimens comprised of only three, primarily Group A drugs rather than the recommended four drugs performed exceedingly well, with >90% culture conversion at six months. These findings point to the possibility that three potent drugs may be sufficient in many patients. In addition, that these findings corroborate those of the Phase III trial, and are at odds with the Phase II, raises red flags about the strategy used for evaluating both delamanid and bedaquiline: each was added as a single drug to a background regimen. The relative efficacy of bedaquiline compared to placebo in the Phase IIB trial was even more pronounced than that of delamanid [40]. It is critical to note the extremely poor performance of background regimen plus placebo in the bedaquiline trial: in the placebo plus background regimen, only 9% of participants experienced culture conversion at 8 weeks. In comparison, in the delamanid Phase IIB trial, 54% of control-arm participants experienced this outcome. The improved standard of care likely contributed to the equivocal results in the Phase III trial of delamanid and the present observational study analysis. The confirmatory Phase III trial of bedaquiline has altered the approach, by considering the potential for bedaquiline to contribute to treatment shortening and bedaquiline as a replacement for a toxic, injectable drug, kanamycin (ClinicalTrials.gov Identifier: NCT02409290). As RR/MDR-TB treatment effectiveness increases, quantifying the contribution of any singular drug will become increasingly difficult, underscoring the importance of evaluating regimens, rather than individual drugs, an approach adopted by several recent pivotal trials [41, 42].

This study highlights important considerations for investigators analyzing MDR-TB treatment cohorts. Analyses of baseline regimen compositions might not answer the most relevant clinical question when regimen composition commonly changes throughout the course of treatment [43].

We did not identify a clinically meaningful difference between results of standard baseline-adjusted models—which estimates the aITT—and the inverse probability censoring weighted analysis estimating the aPP. This tells us that, in this cohort, the effect of adding delamanid to a three-drug regimen at baseline is similar to that of adding and maintaining delamanid in a three-drug regimen for the first six months of treatment. The likely reason for not identifying differences between these analyses is that among patients who changed their regimen, changes to the primary drug of interest, delamanid, were rare (13/98, 13.3%). Only one patient discontinued delamanid due to an adverse event, reinforcing the safety of delamanid and potential use of delamanid in regimens compromised by toxicity. Changes in the number of likely effective background drugs in the background regimen were more common; however, meaningful differences between estimates will be observed if censoring (i.e., change in the baseline regimen) is highly (many-fold) associated with both the treatment group (i.e., delamanid at baseline) and the outcome. This was not the case in this analysis (Appendix E in S1 Text) [43, 44]. While we did not observe a meaningful difference between *a*ITT and *a*PP estimates, when the objective is to estimate the effect of starting and remaining on a treatment, the approach applied here is one strategy that also resolves the potential for time-dependent confounding [37]. Target trial emulation is an intuitive framework to assist investigators through the steps of identifying the research question, the treatment groups to be compared, and an analytic approach that will produce an estimate of the desired causal effect [26].

A limitation of this analysis is that, despite narrowly defining our treatment groups, and adjusting for the number of Group A drugs, there may be residual differences in the quality of the background regimen across the groups. There was an imbalance of the number Group A drugs in the three-drug regimen without delamanid (92.4% had 2 or 3 Group A drugs) and three-drug regimen plus delamanid (58% had 2 or 3 Group A drugs). Few efforts have been made to meaningfully capture the heterogeneity of individualized MDR-TB regimens in comparative effectiveness studies, likely because hundreds or even thousands of distinct regimens can be represented in any one cohort. For example, the 2012 individual patient data meta-analysis comprises over 9000 patients on 1626 different baseline regimens [45]. Further methodologic work in this area is needed, as global treatment guidance relies largely on observational cohorts for the evidence base [45–47]. Unmeasured confounding by non-regimen factors (patient-level factors such as demographics or disease severity) is also possible, though less likely because we collected and adjusted for a multitude of baseline and time-varying factors. Lastly, culture conversion is an imperfect predictor of final treatment outcome, therefore we cannot conclude the association of delamanid with the proportion cured.

In conclusion, although, we did not identify a benefit for 2- or 6- month culture conversion of adding delamanid to an MDR/RR-TB regimen with only three likely effective, and often highly-potent drugs, the rarity of delamanid suspension reinforces existing evidence about its safety. Important questions remain about how to optimize the use of delamanid, including whether delamanid can improve the effectiveness of regimens comprised of drugs with suboptimal efficacy or improve the safety (or efficacy) of treatment through substitution for more toxic (less potent) agents. Our findings also highlight the risk of equivocal results if the drug-development approach used for bedaquiline and delamanid is applied to new chemical entities in the context of the current, improved background regimen. Finally, the analytic methods used here can facilitate articulation of precise research questions and should be considered as a strategy for reducing bias in analysis of MDR/RR-TB regimens, when treatment changes are frequent.

## Supporting information

S1 Text (DOCX)

## References

[1] World Health Organization. Global Tuberculosis Report 2022. Geneva, Switzerland; 2022.

[2] World Health Organization. Global Tuberculosis Report 2020. Geneva, Switzerland; 2020.

[3] Bloss E, Kukša L, Holtz TH, Riekstina V, Skripčonoka V, Kammerer S, et al. Adverse events related to multidrug-resistant tuberculosis treatment, Latvia, 2000–2004. Int J Tuberc Lung Dis. 2010;14(3):275–81. 20132617

[4] Cox HS, Kalon S, Allamuratova S, Sizaire V, Tigay ZN, Rüsch-Gerdes S, et al. Multidrug-resistant tuberculosis treatment outcomes in Karakalpakstan, Uzbekistan: Treatment complexity and XDR-TB amont treatment failures. PLoS One. 2007;2(11):e1126. 17987113 10.1371/journal.pone.0001126 PMC2040509

[5] World Health Organization. WHO consolidated guidelines on tuberculosis. Module 4: treatment—drug-resistant tuberculosis treatment. Geneva, Switzerland; 2022. 36630546

[6] European Medicines Agency. European Medicines Agency recommends two new treatment options for tuberculosis [Internet]. Press Release. 2013 [cited 2022 Apr 15]. Available from: http://www.ema.europa.eu/ema/index.jsp?curl=pages/news_and_events/news/2013/11/news_detail_001972.jsp&mid=WC0b01ac058004d5c1

[7] Gler MT, Skripconoka V, Sanchez-Garavito E, Xiao H, Cabrera-Rivero JL, Vargas-Vasquez DE, et al. Delamanid for Multidrug-Resistant Pulmonary Tuberculosis. N Engl J Med. 2012;366(23):2151–60. doi: 10.1056/NEJMoa1112433 22670901

[8] von Groote-Bidlingmaier F, Patientia R, Sanchez E, Balanag VJ, Ticona E, Segura P, et al. Efficacy and safety of delamanid in combination with an optimised background regimen for treatment of multidrug-resistant tuberculosis: a multicentre, randomised, double-blind, placebo-controlled, parallel group phase 3 trial. Lancet Respir Med. 2019;7(3):249–59. doi: 10.1016/S2213-2600(18)30426-0 30630778

[9] Nahid P, Mase SR, Migliori GB, Sotgiu G, Bothamley GH, Brozek JL, et al. Treatment of drug-resistant tuberculosis an official ATS/CDC/ERS/IDSA clinical practice guideline. Am J Respir Crit Care Med. 2019;200(10):E93–142. 31729908 10.1164/rccm.201909-1874ST PMC6857485

[10] World Health Organization. WHO treatment guidelines for rifampicin- and multidrug-resistant tuberculosis, 2018 update. Geneva, Switzerland; 2018.

[11] World Health Organization. Rapid communication: key changes to treatment of multidrug- and rifampicin-resistant tuberculosis (MDR/RR-TB). Geneva, Switzerland; 2018.

[12] Hafkin J, Hittel N, Martin A, Gupta R. Early outcomes in MDR-TB and XDR-TB patients treated with delamanid under compassionate use. Eur Resp J. 2017;50(1):1700311. doi: 10.1183/13993003.00311-2017 28751415 PMC5898945

[13] Ferlazzo G, Mohr E, Laxmeshwar C, Hewison C, Hughes J, Jonckheere S, et al. Early safety and efficacy of the combination of bedaquiline and delamanid for the treatment of patients with drug-resistant tuberculosis in Armenia, India, and South Africa: a retrospective cohort study. Lancet Infect Dis. 2018;18(5):536–44. doi: 10.1016/S1473-3099(18)30100-2 29452942

[14] Ghosh S, Breitscheidel L, Lazarevic N, Martin A, Hafkin J, Hittel N. Compassionate use of delamanid in adults and children for drug-resistant tuberculosis: 5-year update. Eur Respir J [Internet]. 2021 May 1 [cited 2023 Feb 15];57(5). Available from: https://pubmed.ncbi.nlm.nih.gov/33243846/ doi: 10.1183/13993003.02483-2020 33243846

[15] Kwon YS, Jeon D, Kang H, Yim JJ, Shim TS. Concurrent use of bedaquiline and delamanid for the treatment of fluoroquinolone-resistant multidrug-resistant tuberculosis: A nationwide cohort study in South Korea. Eur Resp J. 2021;57(3):2003026. 10.1183/13993003.03026-2020 33093123

[16] Das M, Dalal A, Laxmeshwar C, Ravi S, Mamnoon F, Meneguim AC, et al. One Step Forward: Successful End-of-Treatment Outcomes of Patients with Drug-Resistant Tuberculosis Who Received Concomitant Bedaquiline and Delamanid in Mumbai, India. Clin Infect Dis. 2021;73(9):E3496–504. doi: 10.1093/cid/ciaa1577 33079176

[17] Kang H, Jo KW, Jeon D, Yim JJ, Shim TS. Interim treatment outcomes in multidrug-resistant tuberculosis using bedaquiline and/or delamanid in South Korea. Respir Med. 2020;167:105956. 32421540 10.1016/j.rmed.2020.105956

[18] Khan U, Huerga H, Khan AJ, Mitnick CD, Hewison C, Varaine F, et al. The endTB observational study protocol: Treatment of MDR-TB with bedaquiline or delamanid containing regimens. BMC Infect Dis. 2019;19(1). doi: 10.1186/s12879-019-4378-4 31429722 PMC6701145

[19] World Health Organization. The use of bedaquiline in the treatment of multidrug-resistant tuberculosis interim policy guidance. Geneva, Switzerland: World Health Organization; 2013. 23967502

[20] World Health Organization. The use of delamanid in the treatment of multidrug-resistant tuberculosis interim policy guidance. Geneva: World Health Organization; 2014. 26110189

[21] endTB Consortium. endTB Clinical and Programmatic Guide for Patient Management with New TB Drugs. Version 4.0. 2018.

[22] Lachenal N, Hewison C, Mitnick C, Lomtadze N, Coutisson S, Osso E, et al. Setting up pharmacovigilance based on available endTB Project data for bedaquiline. Int J Tuberc Lung Dis. 2020;24(10):1087–94. doi: 10.5588/ijtld.20.0115 33126944

[23] Caniglia EC, Zash R, Jacobson DL, Diseko M, Mayondi G, Lockman S, et al. Emulating a target trial of antiretroviral therapy regimens started before conception and risk of adverse birth outcomes. AIDS. 2018;32(1):113–20. doi: 10.1097/QAD.0000000000001673 29112066 PMC5718935

[24] Labrecque JA, Swanson SA. Target trial emulation: teaching epidemiology and beyond. Eur J Epidemiol. 2017;32(6):473–5. doi: 10.1007/s10654-017-0293-4 28770358 PMC5550532

[25] Hernán MA, Sauer BC, Hernández-Díaz S, Platt R, Shrier I. Specifying a target trial prevents immortal time bias and other self-inflicted injuries in observational analyses. J Clin Epidemiol. 2016;79:70–5. doi: 10.1016/j.jclinepi.2016.04.014 27237061 PMC5124536

[26] Hernán MA, Robins JM. Using Big Data to Emulate a Target Trial When a Randomized Trial Is Not Available. Am J Epidemiol. 2016 Apr 15;183(8):758–64. doi: 10.1093/aje/kwv254 26994063 PMC4832051

[27] García-Albéniz X, Hsu J, Hernán MA. The value of explicitly emulating a target trial when using real world evidence: an application to colorectal cancer screening. Eur J Epidemiol. 2017;32(6):495–500. doi: 10.1007/s10654-017-0287-2 28748498 PMC5759953

[28] Trevisi L, Hernán MA, Mitnick CD, Khan U, Seung KS, Rich ML, et al. Effectiveness of bedaquiline use beyond six months in patients with multidrug-resistant tuberculosis. Am J Respir Crit Care Med. 2023. In Press. doi: 10.1164/rccm.202211-2125OC 36802336 PMC10263131

[29] Kurbatova E V, Cegielski JP, Lienhardt C, Akksilp R, Bayona J, Becerra MC, et al. Sputum culture conversion as a prognostic marker for end-of-treatment outcome in patients with multidrug-resistant tuberculosis: a secondary analysis of data from two observational cohort studies. Lancet Respir Med. 2015;3(3):201–9. doi: 10.1016/S2213-2600(15)00036-3 25726085 PMC4401426

[30] Meyvisch P, Kambili C, Andries K, Lounis N, Theeuwes M, Dannemann B, et al. Evaluation of six months sputum culture conversion as a surrogate endpoint in a multidrug resistant-tuberculosis trial. PLoS One. 2018;13(7):e0200539–e0200539. doi: 10.1371/journal.pone.0200539 30024924 PMC6053142

[31] Rodriguez CA, Lodi S, Horsburgh CR, Bastard M, Hewison C, Huerga H, et al. Selection bias in multidrug-resistant tuberculosis cohort studies assessing sputum culture conversion. PLoS One. 2022;17(11):e0276457. doi: 10.1371/journal.pone.0276457 36355658 PMC9648724

[32] Rodriguez CA, Brooks MB, Aibana O, Mitnick CD, Franke MF. Sputum culture conversion definitions and analytic practices for multidrug-resistant TB. Int J Tuberc Lung Dis. 2021;25(7):596–8. doi: 10.5588/ijtld.21.0090 34183109 PMC8259120

[33] Rodriguez CA, Sy KTL, Mitnick CD, Franke MF. Time-Dependent Confounding in Tuberculosis Treatment Outcome Analyses: A Review of a Source of Bias. Am J Respir Crit Care Med. 2020;202(9):1311–4. doi: 10.1164/rccm.202001-0220LE 32551891

[34] Naimi AI, Cole SR, Kennedy EH. An introduction to g methods. Int J Epidemiol. 2017;46(2):756–62. doi: 10.1093/ije/dyw323 28039382 PMC6074945

[35] Daniel RM, Cousens SN, De Stavola BL, Kenward MG, Sterne JAC. Methods for dealing with time-dependent confounding. Stat Med. 2013;32(9):1584–618. doi: 10.1002/sim.5686 23208861

[36] Muller CJ, MacLehose RF. Estimating predicted probabilities from logistic regression: different methods correspond to different target populations. Int J Epidemiol. 2014;43(3):962–70. doi: 10.1093/ije/dyu029 24603316 PMC4052139

[37] Hernán MA, Lanoy E, Costagliola D, Robins JM. Comparison of dynamic treatment regimes via inverse probability weighting. Basic Clin Pharmacol Toxicol, 2006;98(3):237–42. doi: 10.1111/j.1742-7843.2006.pto_329.x 16611197

[38] Franke MF, Mitnick CD. Time for a change: considering regimen changes in analyses of observational drug-resistant TB treatment cohort data. Int J Tuberc Lung Dis. 2020;24(11):1151–5. doi: 10.5588/ijtld.20.0076 33172522

[39] Rich ML, Khan U, Zeng C, LaHood A, Franke MF, Atwood S, et al. Outcomes of WHO-conforming longer all-oral multidrug-resistant tuberculosis regimens & analysis implications. Int J Tuberc Lung Dis. 2023; In Press. 10.5588/ijtld.22.0613 PMC10237267 37231598

[40] Diacon AH, Pym A, Grobusch MP, de los Rios JM, Gotuzzo E, Vasilyeva I, et al. Multidrug-Resistant Tuberculosis and Culture Conversion with Bedaquiline. New England Journal of Medicine. 2014;371(8):723–32. doi: 10.1056/NEJMoa1313865 25140958

[41] Guglielmetti L, Ardizzoni E, Atger M, Baudin E, Berikova E, Bonnet M, et al. Evaluating newly approved drugs for multidrug-resistant tuberculosis (endTB): study protocol for an adaptive, multi-country randomized controlled trial. Trials. 2021;22(1):651. doi: 10.1186/s13063-021-05491-3 34563240 PMC8465691

[42] Nyang’wa BT, Berry C, Kazounis E, Motta I, Parpieva N, Tigay Z, et al. A 24-Week, All-Oral Regimen for Rifampin-Resistant Tuberculosis. N Engl J Med. 2022 21;387(25):2331–43. doi: 10.1056/NEJMoa2117166 36546625

[43] Keyes KM, Jager J, Platt J, Rutherford C, Patrick ME, Kloska DD, et al. When does attrition lead to biased estimates of alcohol consumption? Bias analysis for loss to follow-up in 30 longitudinal cohorts. Int J Methods Psychiatr Res. 2020;29(4):e1842. doi: 10.1002/mpr.1842 32656917 PMC7723204

[44] Weuve J, Tchetgen Tchetgen EJ, Glymour MM, Beck TL, Aggarwal NT, Wilson RS, et al. Accounting for bias due to selective attrition: the example of smoking and cognitive decline. Epidemiology. 2012;23(1):119–28. doi: 10.1097/EDE.0b013e318230e861 21989136 PMC3237815

[45] Ahuja SD, Ashkin D, Avendano M, Banerjee R, Bauer M, Bayona JN, et al. Multidrug resistant pulmonary tuberculosis treatment regimens and patient outcomes: an individual patient data meta-analysis of 9,153 patients. PLoS Med. 2012;9(8):e1001300. doi: 10.1371/journal.pmed.1001300 22952439 PMC3429397

[46] Ahmad N, Ahuja SD, Akkerman OW, Alffenaar JWC, Anderson LF, Baghaei P, et al. Treatment correlates of successful outcomes in pulmonary multidrug-resistant tuberculosis: an individual patient data meta-analysis. The Lancet. 2018;392(10150):821–34. doi: 10.1016/S0140-6736(18)31644-1 30215381 PMC6463280

[47] Siddique AA, Schnitzer ME, Bahamyirou A, Wang G, Holtz TH, Migliori GB, et al. Causal inference with multiple concurrent medications: A comparison of methods and an application in multidrug-resistant tuberculosis. Stat Methods Med Res. 2018;28(12):3534–49. doi: 10.1177/0962280218808817 30381005 PMC6511477

