# Supplementary material for: Comparative effectiveness of adding delamanid to a multidrug-resistant tuberculosis regimen comprised of three drugs likely to be effective
Source: PLOS Glob Public Health. 2023 Apr 28;3(4):e0000818. doi: 10.1371/journal.pgph.0000818 (PMC10146539; doi:10.1371/journal.pgph.0000818)
Supplement: S1 Text — (DOCX) [file pgph.0000818.s001.docx]

Table of Contents

[Appendix A. Target trial protocol and emulation of the target trial using endTB observational cohort data 1](#_Toc100911922)

[Appendix B. Model specification and weight diagnostics for inverse probability of censoring weights 3](#_Toc100911923)

[Appendix C. Derivation of censoring weights 4](#_Toc100911924)

[Appendix D. Baseline treatment regimens of participants receiving three drugs likely to be effective +/- delamanid, endTB observational cohort (N=362) 5](#_Toc100911925)

[Appendix E. Descriptive analysis of the distribution of censoring 7](#_Toc100911926)

[Table A. Proportion of participants censored, by treatment group and two- and six-month culture conversion in analysis to assess the effect of adding delamanid to a regimen composed of three drugs likely to be effective, endTB observational cohort (N=362) 8](#_Toc100911927)

[Table B. Crude association of censoring with treatment group and outcome in analysis to assess the effect of adding delamanid to a regimen composed of three drugs likely to be effective, endTB observational cohort (N=362) 9](#_Toc100911928)

# Appendix A. Target trial protocol and emulation of the target trial using endTB observational cohort data

Objective: Evaluate the effect of adding delamanid to an MDR-TB regimen that contains 3 likely effective drugs.

|  | **Target trial** | **Observational analysis (endTB observational cohort)** |
| --- | --- | --- |
| **Eligibility criteria** | **Inclusion**   - Age 14+ - Diagnosis of MDR-TB from: 1) a sputum specimen collected no more than 90 days prior to the date of trial screening with positive growth of *M.tb* in culture and documented phenotypic resistance to rifampin OR 2) a sputum specimen collected no more than 90 days prior to the date of trial screening with positive growth of *M.tb* in culture, and a genotypic test positive for *M.tb* and with mutations known to confer resistance to rifampin | Same |
| **Treatment strategies** | **Intervention**  Delamanid plus a background regimen of 3 likely effective drugs administered for 24 weeks, where a likely effective drug is defined as a drug for which resistance testing indicated participants’ *M.tb* strain was not resistant to the drug or a drug for which no resistance testing was conducted and the participant had not previously received the drug for ≥1 month  **Comparator**  A regimen of 3 likely effective drugs, none of which are delamanid, administered for 24 weeks | Same, but baseline exposure determined as treatment regimen on 7^th^ day of treatment |
| **Treatment assignment** | Randomized | Randomization assumed within levels of covariates |
| **Follow up** | **Time zero**  Treatment assignment  **End of follow up**  Outcome, 24 weeks after time zero  **LTFU**  Treatment interruption for ≥2 months | **Time zero**  The earliest start date of bedaquiline or delamanid initiation with endTB  **End of follow up**  Same  **LTFU**  Same |
| **Outcome** | Six-month culture conversion, defined as two, consecutive negative cultures collected at least 15 days apart, the first occurring before 180 days of treatment and the second before 210 days; death and LTFU prior to 180 days are considered non-conversion events. | Same |
| **Causal contrast** | Intention-to-treat effect | Per-protocol effect |
| **Statistical analysis** | Crude ratio and difference of the proportion with two-month and six-month culture conversion across arms  **Main effect measure**  Two-month culture conversion risk ratio and risk difference  Six-month culture conversion risk ratio and risk difference | Inverse probability censoring weights  Patients are censored when they deviate from the baseline (day 7 exposure) unless it is a discontinuation of delamanid due to adverse event OR if the deviation was for ≤2 weeks consecutive duration  **Main effect measure**  Same |

# Appendix B. Model specification and weight diagnostics for inverse probability of censoring weights

| **Model** | **Specification** | **Weight** | **N** | **Mean (SD)** | **Min, max** |
| --- | --- | --- | --- | --- | --- |
| **Primary** | Numerator includes time (stabilized)· Denominator includes exposure group, time-varying linear term for # Group A drugs, time-varying smear result (+/-), time-varying linear term for # AEs, time-varying term for hospitalization (0/1), a 3-knot spline for time, time· | Unstabilized | 348^a^ | 1·37 (0·49) | 1·00, 6·44 |
|  |  | Stabilized | 348^a^ | 0·99 (0·34) | 0·75, 4·60 |
| **Sensitivity: BDQ** | Numerator includes time (stabilized)· Denominator includes exposure group, time-varying linear term for # Group A drugs, time-varying smear result (+/-), time-varying linear term for # AEs, time-varying term for hospitalization (0/1), a 3-knot spline for time, time | Unstabilized | 288^b^ | 1·33 (0·52) | 1·00, 7·22 |
|  |  | Stabilized | 288^b^ | 1·00 (0·38) | 0·77, 5·36 |

**Abbreviations:** Standard deviation (SD), adverse event (AE), serious adverse event (SAE), Sensitivity analysis restricted to participants also receiving bedaquiline (BDQ)

^b^ N=10 participants excluded for missing time-varying data, N=2 participants excluded for missing baseline and time-varying data, N=2 participants excluded for missing baseline data

^c^ N=7 participants excluded for missing time-varying data, N=1 participant excluded for missing baseline data

# Appendix C. Derivation of censoring weights

For unstabilized censoring weights (W^C^), we divided 1 by the probability of a participant remaining on their baseline treatment regimen using the formula below where: *t* denotes the time in weeks from treatment initiation to the time-fixed culture conversion endpoint (2 months, 6 months, death or LTFU), A is the baseline exposure (1=delamanid-containing, 0=delamanid-free), C(*t*) indicates artificial censoring due to switching exposure groups at time *t* (1= censored, 0=not censored), $\bar{L}$(*t*) represents the vector of time-varying covariates at time *t* and L represents the vector of baseline covariates used to model time-varying covariate history. At time *t*, the denominator of the weight is the product of the predicted probability of being uncensored.

W^C^ (*t*) = $\prod_{k=0}^{t} \frac{1}{Pr[C\left( k \right)=0│L,\bar{L}\left( k \right), C\left( k-1 \right)=0, A]}$

For statistical efficiency of our risk estimate, we additionally derived stabilized censoring weights (SW^C^) by fitting a second pooled logistic regression to estimate the probability of the participant remaining on their baseline treatment regimen (i.e. not being censored), conditional on time, as represented by the following formula:

SW^C^ (*t*) = $\prod_{k=0}^{t} \frac{Pr(C\left( k \right)=0│C\left( k-1 \right)=0, A)}{Pr[C\left( k \right)=0│L,\bar{L}\left( k \right), C\left( k-1 \right)=0, A]}$

We fitted multiple models, identifying predictors of treatment group changes *a priori* based on content knowledge. The mean and standard deviation of estimated weights are described in Appendix B.

# Appendix D. Baseline treatment regimens of participants receiving three drugs likely to be effective +/- delamanid, endTB observational cohort (N=362)

| **Baseline regimen** | **Delamanid containing^a^ (N=123)** | **Delamanid-free**  **(N=239)** |
| --- | --- | --- |
|  | **n** (%) | **n** (%) |
| **3 Group A drugs^b^** | | |
| Lfx/Mfx, Bdq, Lzd | 0 (-) | 24 (0·1) |
| **2 Group A drugs^b^** | | |
| Bdq, Lzd, Cfz | 48 (0·39) | 142 (0·59) |
| Bdq, Lzd, CilIm/M | 1 (0·01) | 20 (0·08) |
| Bdq, Lzd, Csn/Trd | 1 (0·01) | 16 (0·07) |
| Bdq, Lzd, E | 0 (-) | 1 (0) |
| Bdq, Lzd, Eto/Pto | 1 (0·01) | 1 (0) |
| Bdq, Lzd, Km/Cm | 0 (-) | 4 (0·02) |
| Bdq, Lzd, PASms/PA | 0 (-) | 4 (0·02) |
| Lfx/Mfx, Bdq, Cfz | 1 (0·01) | 4 (0·02) |
| Lfx/Mfx, Bdq, Csn/Trd | 0 (-) | 3 (0·01) |
| Lfx/Mfx, Bdq, E | 0 (-) | 1 (0) |
| Lfx/Mfx, Bdq, Km/Cm | 1 (0·01) | 0 (-) |
| Lfx/Mfx, Bdq, PASms/PA | 0 (-) | 1 (0) |
| Lfx/Mfx, Lzd, Cfz | 12 (0·1) | 0 (-) |
| Lfx/Mfx, Lzd, CilIm/M | 2 (0·02) | 0 (-) |
| Lfx/Mfx, Lzd, Csn/Trd | 2 (0·02) | 0 (-) |
| Lfx/Mfx, Lzd, Eto/Pto | 2 (0·02) | 0 (-) |
| Lfx/Mfx, Lzd, Km/Cm | 1 (0·01) | 0 (-) |
| **1 Group A drug^b^** | | |
| Bdq, Cfz, CilIm/M | 3 (0·02) | 6 (0·03) |
| Bdq, Cfz, Eto/Pto | 0 (-) | 1 (0) |
| Bdq, Cfz, Km/Cm | 0 (-) | 5 (0·02) |
| Bdq, Cfz, PASms/PA | 0 (-) | 1 (0) |
| Bdq, Cfz, S/A | 0 (-) | 2 (0·01) |
| Bdq, CilIm/M, Eto/Pto | 0 (-) | 1 (0) |
| Bdq, Csn/Trd, Eto/Pto | 0 (-) | 1 (0) |
| Bdq, Csn/Trd, PASms/PA | 1 (0·01) | () |
| H, Bdq, CilIm/M | 0 (-) | 1 (0) |
| Lfx/Mfx, Cfz, CilIm/M | 1 (0·01) | 0 (-) |
| Lfx/Mfx, Cfz, Eto/Pto | 1 (0·01) | 0 (-) |
| Lfx/Mfx, Csn/Trd, Eto/Pto | 7 (0·06) | 0 (-) |
| Lzd, Cfz, CilIm/M | 26 (0·21) | 0 (-) |
| Lzd, Cfz, E | 1 (0·01) | 0 (-) |
| Lzd, Cfz, Km/Cm | 2 (0·02) | 0 (-) |
| Lzd, Cfz, PASms/PA | 2 (0·02) | 0 (-) |
| Lzd, Cfz, S/A | 2 (0·02) | 0 (-) |
| Lzd, Cfz, Z | 1 (0·01) | 0 (-) |
| Lzd, CilIm/M, Km/Cm | 1 (0·01) | 0 (-) |
| Lzd, Csn/Trd, PASms/PA | 1 (0·01) | 0 (-) |
| **No Group A drugs^b^** | | |
| Cfz, Csn/Trd, Eto/Pto | 1 (0·01) | 0 (-) |
| Lfx/Mfx, Cfz, Z, Eto/Pto | 1 (0·01) | 0 (-) |

**Abbreviations:** bedaquiline (Bdq), clofazimine (Cfz), cycloserine or terizidone (Csn/Tzd), ethambutol (E), ethionamide or prothionamide (Eto/Pto), imipenem-cilastatin-amoxicillin clavulanate or meropenem (ImCil/M), levofloxacin or moxifloxacin (Lfx/Mfx), linezolid (Lzd), p-aminosalicylic acid (PAS), pyrazinamide (Z), streptomycin or amikacin (S/A)

^a^ All participants on a delamanid-containing regimen also received delamanid (not shown in regimen list)

^b^ Group A drugs include bedaquiline (Bdq), linezolid (Lzd), and levofloxacin or moxifloxacin (Lfx/Mfx)

# Appendix E. Descriptive analysis of the distribution of censoring

To investigate the degree to which treatment varied over time, we assessed the distribution of censoring in the data according to baseline treatment group and each outcome. We additionally calculated crude relative risks to identify the association between censoring and the treatment group (delamanid-containing regimens) and each outcome using the following formula, where C=1 represents the number of participants censored and Y=1 represents the number of participants on delamanid-containing regimens or that converted: $\frac{\frac{C=1, Y=1}{\left( C=1,Y=1 \right)+(C=0, Y=1)}}{\frac{C=1,Y=0}{\left( C=1,Y=0 \right)+(C=0,Y=0)}}$.

As reported in the main text, 98 (27·0%) participants were artificially censored because their regimen was changed such that it resulted in a treatment group switch for more than two weeks (Figure 2). Censored participants were nearly proportionally represented within their respective baseline treatment groups (30·1% censored in delamanid-containing, 25·1% censored in delamanid-free). Receiving a delamanid-containing regimen was not strongly associated with changing treatment groups (i.e. censoring) (crude RR: 1·68 (95% CI: 1·27, 2·24). Participants who converted were less likely to have changed treatment groups (i.e. be censored) than those who did not experience conversion (crude RR at two months: 0·71 (95% CI: 0·55, 0·92); crude RR at six months: 0·90 (95% CI: 0·80, 1·00)).

# Table A. Proportion of participants censored, by treatment group and two- and six-month culture conversion in analysis to assess the effect of adding delamanid to a regimen composed of three drugs likely to be effective, endTB observational cohort (N=362)

|  | **Delamanid-containing (N=123)** | | **Delamanid-free (N=239)** | |
| --- | --- | --- | --- | --- |
|  | **Censored** | **Uncensored** | **Censored** | **Uncensored** |
| **Total, n/N (%)** | 37/123 (30·1) | 86/123 (69·9) | 60/239 (25·1) | 179/239 (74·9) |
| Two-month, converted, n/N (%) | 15/37 (40·5) | 46/86 (53·5) | 25/60 (41·7) | 108/179 (60·3) |
| Two-month, did not convert, n/N (%) | 22/37 (59·4) | 40/86 (46·5) | 35/60 (58·3) | 71/179 (39·7) |
| Six-month, converted, n/N (%) | 29/37 (78·4) | 71/86 (82·6) | 48/60 (80·0) | 164/179 (91·6) |
| Six-month, did not convert, n/N (%) | 8/37 (21·6) | 15/86 (17·4) | 12/60 (20·0) | 15/179 (8·4) |

# Table B. Crude association of censoring with treatment group and outcome in analysis to assess the effect of adding delamanid to a regimen composed of three drugs likely to be effective, endTB observational cohort (N=362)

| **Association** | **Risk ratio (95% CI)** |
| --- | --- |
| Association between censoring and treatment group | 1·68 (1·27, 2·24) |
| Association between censoring and two-month conversion | 0·71 (0·55, 0·92) |
| Association between censoring and six-month conversion | 0·90 (0·80, 1·00) |
